# Cytotoxic, antioxidative, genotoxic and antigenotoxic effects of *Horchata*, beverage of South Ecuador

**DOI:** 10.1186/s12906-017-2048-x

**Published:** 2017-12-19

**Authors:** Natalia Bailon-Moscoso, Fani Tinitana, Ruth Martínez-Espinosa, Andrea Jaramillo-Velez, Alejandra Palacio-Arpi, Jessica Aguilar-Hernandez, Juan Carlos Romero-Benavides

**Affiliations:** 1 Departamento de Ciencias de la Salud, Universidad Técnica Particular de Loja, Loja, Ecuador; 2 Departamento de Ciencias Biológicas, Universidad Técnica Particular de Loja, Loja, Ecuador; 3 Departamento de Química y Ciencias Exactas, Universidad Técnica Particular de Loja, Loja, Ecuador

**Keywords:** *Horchata*, Cancer, Antigenotoxicity, Anticlastogenic, Apoptosis

## Abstract

**Background:**

“Horchata” is an herbal mixture infusion consumed in Southern Ecuador; 66% of its plants are anti-inflammatory medicinal plant, and 51% are analgesics. Anti-inflammatory substances can prevent carcinogenesis mediated by cytotoxic effects and can prevent DNA damage. The aim of this study was to evaluate the cytotoxicity and apoptotic/antigenotoxic effects of horchata as well as its mechanism.

**Methods:**

Nine different varieties of horchata were prepared in the traditional way and then freeze-dried. Phytochemical screening tested for the presence of secondary metabolites using standard procedures and antioxidant activities. The cytotoxic activity was evaluated on cerebral astrocytoma (D-384), prostate cancer (PC-3), breast cancer (MCF-7), colon cancer (RKO), lung cancer (A-549), immortalized Chinese hamster ovary cells (CHO-K1), and human peripheral blood lymphocytes via a MTS assay. The pro-apoptotic effects were evaluated with Anexin V/Propidium Iodide and western blot of Bax, Bcl-2, TP53, and TP73. Induction and reduction of ROS were assessed by fluorimetry. Genotoxic and antigenotoxic effects were evaluated with a comet assay and micronuclei on binucleated cells.

**Results:**

Five of nine horchatas had cytotoxic effects against D-384 while not affecting normal cells. These horchatas induce cell death by apoptosis modulated by p53/p73. In CHO-K1 cells, the horchatas decrease the damage induced by hydrogen peroxide and Mitomycin C measured in the comet and micronucleus assay respectively.

**Conclusions:**

The IC_50_ range of effective horchatas in D-384 was 41 to 122 μg·mL^−1^. This effect may be related to its use in traditional medicine (brain tonic). On the other hand, immortalized Chinese hamster ovary cells (CHO-K1) and lymphocytes did not show a cytotoxic effect. The most potent horchata induced apoptosis via a p53/p73-mediated mechanism.

The horchatas present antigenotoxic properties, which may be related to the antioxidant capacity. Future studies on horchata components are necessary to understand the interactions and beneficial properties.

## Background

The low incidence of chronic non-communicable diseases such as cardiovascular diseases, metabolic disorders, and lower risk of a variety of cancers is associated with frequent consumption of plant-derived foods such as vegetables, fruits, tea, and herbs [1]. “Horchata” (HRCH) is a heritage drink in Southern Ecuador and is consumed for its therapeutic uses. HRCH is also called “aguas frescas” or “agua de frescos” and is an herbal mixture infusion of 16 to 32 medicinal plants depending on their spatial and temporal availability. The species to prepare HRCH are generally traded in a group locally named as “atado”, “manojo”, “ramillete” or “tongo” that is boiled for 10 min and can be consumed cold or hot [2].

HRCH is very popular in in Southern Ecuador particularly among the indigenous people who believe that the therapeutic effect of the drink is improved when plants locally known as “calientes” (warm) and “frías” (cold) are properly combined. The urban and rural people who consume horchata in Loja province report positive benefits.

Previous studies have shown that some culturally important plant species can treat circulatory, digestive, nervous, and respiratory disease; 66% of medicinal plant species are anti-inflammatory, and 51% are analgesics [2]. These activities are related to protective effects because these chronic inflammation processes are related to carcinogenesis [3–8], while anti-inflammatory substances contribute to chemoprevention [9–14]. Cancer chemoprevention includes long-term administration of pharmacological or natural compounds to prevent or slow down the cancer development [15–19]. Antigenotoxic agents exhibit desirable chemotherapeutic effects and control cancer.

Studies of cytotoxicity and genotoxicity/antigenotoxicity are rapid methods to assess the innocuousness and possible beneficial effects of single compounds or complex mixtures and foods [20, 21, 33, 34]. The aim of this study is to evaluate the protective effects of this drink in southern Ecuador. We studied the cytotoxic capacity in tumor cells as a protector of DNA damage using genotoxic agents that follow different DNA damaging pathways including oxidation and alkylation.

## Methods

### Materials

The following reagents and chemicals were purchased from Sigma-Aldrich, St. Louis, USA: 2′-7′-dichlorofluorescin diacetate (H_2_DCFDA), balanced salt solution (HBSS), sodium hydroxide (NaOH), potassium chloride (KCl), sodium bicarbonate (NaHCO_3_), sodium phosphate dibasic anhydrous (NaHPO_4_), monobasic potassium phosphate (KH_2_PO_4_), disodium ethylenediaminetetraacetate dihydrate (Na_2_EDTA), dimethyl sulfoxide (DMSO), sodium chloride (NaCl), methanol, ethanol, peroxidemitomycin C, doxurrubicin, (±)-6 hydroxy-2,5,7,8 tetra-methylchromane-2-carboxylic acid (Trolox), 2 diphenyl-1-picrylhydrazyl (DPPH), 2,4,6, tris(2-pyridyl)-s triazine for spectrophometric determination (of Fe) (TPTZ), iron III chloride hexahydrate, gallic acid, Folin–Ciocalteu’s reagent, and cytochalasin B. The Tris and UltraPure Agarose were purchased from Invitrogen, Carlsbad, California, USA. Roswell Park Memorial Institute (RPMI) 1640 medium, HAM-F12, fetal bovine serum (FBS), L–glutamine, antibiotic-antimycotic (100 units·mL^−1^ penicillin G, 100 μg·mL^−1^ streptomycin and 0.25 μg·mL^−1^ amphotericin B), phytohemagglutinin and formaldehyde were purchased from GIBCO, Grovemont Cir, Gaithersburg, MD USA. Sodium acetate trihydrate, fuming hydrochloric acid, acetic acid (glacial) and sodium carbonate were purchased from Merck KGaA, Darmstadt, Germany. Triton X-100, agarose (LMP), and 3-(4,5-dimethylthiazol-2-yl)-2,5-diphenyltetrazolium (MTS) for CellTiter 96® Aqueous One Solution Cell Proliferation Assay were purchased from Promega, Madison, Wisconsin, USA. Lymphocyte separation medium was purchased from Lonza, Basilea, Suiza. The Anexin-V/IP kit (sc-4252), PVDF transfer membrane (sc-3723), Bcl-2 (C-21, sc-783), Bax (P-19, sc-526), pS46-TP53 (Ser 46, sc-101,764), p-73 (recognizes all p73 isoforms raised against H-79, sc-7957), pY99-TP73 (Tyr 99, sc-101,769), H2AX (C-20, sc-54,606), pS139-H2A.X (Ser 139, sc-517,348), p21 (187, sc-817), β-tubulin (D-10, sc-5274), goat anti-rabbit (sc-2004), and donkey anti-goat (sc-2020) were purchased from Santa Cruz Biotech, San Jose, California, USA. Goat anti-mouse (AP308P) and a chemiluminescence kit (Luminata Crescendo Western HRP substrate) were purchased from EMD-Millipore, Billerica, Massachusetts, USA.

### Lyophilization of the samples

We used samples from the best-selling HRCHs mixtures [2]. Nine different mixtures were prepared, and the compositions are detailed in Table 1. The specimens were purchased from local markets in Loja, Ecuador and identified by Fani Tinitana, PhD. A voucher specimen was deposited in the Herbarium of Universidad Técnica Particular de Loja, Ecuador.

**Table 1:** Species used in different mixtures of HRCHs obtained from markets in Loja city

Family	Species	Common name	Voucher specimen	Samples									Morphological structure used	Therapeutic use	References
				HRCH 1	HRCH 2	HRCH 3	HRCH 4	HRCH 5	HRCH 6	HRCH 7	HRCH 8	HRCH 9			
Adoxaceae	*Sambucus nigra* L*.*	Tilo	FT1252	**X**		**X**	**X**		**X**		**X**		F	Anti-flu, bronquitic, colds, febrifuge, diarrhea and headache.	[13, 14]
Amaranthaceae	*Amaranthus hybridus* L*.*	Ataco	FTMAL006		**X**	**X**			**X**		**X**	**X**	I,L	Anti-inflammatory, anti-flu, anti-hemorrhagic, astringent, healing, diuretic and tonic.	[14]
	*Iresine herbstii* Hook*.*	Escancel	FT0486	**X**	**X**	**X**	**X**	**X**	**X**	**X**	**X**	**X**	F,L,R	Anti-inflammatory, anti-flu, analgesic, diuretic, toning and healing.	[14, 15]
Apiaceae	*Foeniculum vulgare* Mill*.*	Hinojo	FT0025t		**X**			**X**	**X**	**X**			L	Anti-inflammatory, digestive, conjunctivitis, diabetes and used in lactation.	[13, 16]
Asteraceae	*Matricaria recutita* L*.*	Manzanilla	FT0014t	**X**		**X**	**X**	**X**	**X**		**X**	**X**	F,L,S	Anti-inflammatory, anti-flu, anti-flatulence, anthelmintic, febrifuge, digestive, cramps, healing, insomnia. Wounds, burns and stimulating tonic	[14, 17]
Borraginaceae	*Borago officinalis*	Borraja	FT011MAL			**X**	**X**	**X**	**X**		**X**	**X**	F,L	Anti - inflammatory, anti-flu, cough, expectorant, febrifuge, circulation, sudorific, astringent, diuretic and hypocholesterolemiant.	[13, 17–19]
	*Symphytum officinale L.*	Suelda consuelda	FT1084								**X**		L,R	Anti-inflammatory, demulcent, hypotensive, stimulating tissue and nerves, astringent, diuretic and hemostatic.	[17, 20]
Brassicaceae	*Mathiola incana* (L.) W.T. Aiton	Alhelí	FT1250	**X**						**X**			F,L	Anti-inflammatory, anti-flu, sedative, analgesic, cough and digestive.	[13, 14]
Cariophyllaceae	*Dinathus caryophyllus L.*	Clavel	FT1253					**X**		**X**			F,L,P	Anti-inflammatory, analgesic, sedative and cough.	[14, 21]
Equisetaceae	*Equisetum bogotense* Kunth	Cola caballo	FT031t	**X**	**X**	**X**	**X**			**X**	**X**		L,S	Anti-inflammatory, antiseptic, febrifuge and diuretic.	[14, 18]
	*Equisetum giganteun* L.	Cola caballo	FT1009					**X**					L,S	Anti-inflammatory, antiseptic, depurative, hepatic, cancer and cough.	[13, 14]
Geraniaceae	*Pelargonium graveolens L’Her.ex. Aiton*	Malva esencia	FT1258	**X**	**X**	**X**	**X**	**X**	**X**	**X**	**X**	**X**	F,L	Anti-inflammatory, analgesic, febrifuge, diabetes, diarrhea, gallbladder and liver problems, digestive, gastric ulcers, wounds, burns, respiratory diseases, jaundice, infertility, and urinary stones.	[14, 22, 23]
	*Pelargonium odoratissimun (L)* L*’*Hér.	Malva Olorosa	FT016t						**X**	**X**			F,L	Anti-inflammatory, analgesic, carminative and toning.	[14, 23]
Lamiaceae	*Melissa officinalis* L*.*	Toronjil	FT45MPAT	**X**	**X**	**X**			**X**		**X**		L,S	Anti-inflammatory, analgesic, anti-spasmodic, anti-flu, diuretic, ulcerations, digestive, sedative, colds, colic, flatulence, allergies, sudorific, insomnia, heart problems, hemorrhoids and toning.	[14, 19, 24]
	*Mentha x piperita* L*.*	Hierba buena, menta	FT1261					**X**	**X**	**X**		**X**	L,S	Anti-inflammatory, anti-flatulence, febrifuge, liver and gallbladder problems, nerves, cramps, antispasmodic and digestive.	[14, 17, 19]
	*Mentha spicata* L*.*	Menta, hierba buena, menta negra	FT1260			**X**	**X**	**X**	**X**		**X**	**X**	L,S	Anti-inflammatory, anti-espasmodic, anti-flatulence, digestive and colic.	[19, 25]
	*Ocimun basilicum* L*.*	Albahaca negra	FT46TMPA		**X**				**X**				F,L	Anti-inflammatory, anti-espasmodic, anti-flatulence, analgesic cough, headache, ear, digestive, heart problems, nerves, stimulation of lactation and febrifuge.	[14, 19]
	*Ocimum campechianum Mill.*	Albahaca blanca	FTa46TMPA					**X**					F,L	Analgesic, digestive, carminative, febrifuge, ocular cloud, menstrual colic and postpartum bath.	[13, 14]
Linnaceae	*Linum usitatissimum* L*.*	Linaza	FT47TMPA		**X**			**X**	**X**	**X**		**X**	Se	Anti–inflammatory digestive, hepatic and diuretic.	[14, 26]
Malvacea	*Malva arborea (L.) Webb & Berthel.*	Malva blanca, malva alta	FT042MC	**X**	**X**	**X**		**X**	**X**		**X**	**X**	F,L	Depurative, toning and cancer.	[13]
	*Malva parviflora* L*.*	Malva alta,malva blanca	FT015MCE			**X**	**X**		**X**	**X**			F,L	Anti-inflammatory, antidiarrheal, febrifuge, depurative, diuretic, cough, tonic, obesity and insect bites.	[14, 19, 27]
Onagracea	*Fuchsia hybrida* Hort. T. Ex. Siebert & Voss	Pena pena grande, pena pena roja	FT1262					**X**		**X**			P	Anti-inflammatory, anti-flu, sedative, stomachache and toning.	[14]
	*Fuchsia loxensis* Kunth*.*	Pena pena blanca rosada	FT1158	**X**	**X**					**X**			F	Cardiotonic, febrifuge and sedative.	[14]
	*Fuchsia magellanica* Lam.	Pena pena morada	FT0147	**X**	**X**		**X**			**X**			F,L	Sedative.	[14]
	*Oenothera rosea* L’Her. ex Aiton.	Shullo	FT53MPAT	**X**						**X**			F,L,S	Anti-inflamatory, digestive, diuretic and hepatic.	[14, 28]
Piperaceae	*Peperomia inaequalifolia* Ruiz & Pav*.*	Congona grande	FT1197				**X**			**X**			F,L,S	Anti-parasitic, antiperspirant, analgesic, cardiotonic, diuretic, hepatic, sedative earache and insomnia.	[14]
Plantaginaceae	*Plantago major* L*.*	Llanten	FT13t	**X**	**X**	**X**	**X**	**X**	**X**	**X**	**X**		W	Anti-inflammatory, anti- hemorrhagic, digestive liver problems, healing, wounds, insect bites and diuretic.	[14, 19]
Poaceae	*Cymbopogon citratus* (DC) Stapf	Hierba luisa	FT011t	**X**		**X**	**X**	**X**		**X**	**X**	**X**	L	Anti-flatulence, analgesic, digestive, sedative, expectorant, spasmolytic and diuretic.	[14, 29]
Proteaceae	*Oreocallis grandiflora* (Lam.) R.Br.	Cucharillo	FT04t				**X**				**X**		F	Anti-inflammatory, digestive, hepatic astringent and diuretic.	[13, 14]
Rosaceae	*Rosa cymosa* Tratt	Rosa simple rosada blanca	FT274	**X**						**X**			P	Anti-flu, anti-scorbite, digestive, astringent and diuretic.	[30]
Solanaceae	*Solanum americanum* Mill.	Mortiño	FT36t	**X**								X	Fr, L	Anti-inflammatory, analgesic, digestive, febrifuge, sedative and respiratory system.	[14, 31]
Tiliaceae	*Triumfetta semitriloba Jacq.*	Cadillo	FT39t	**X**	**X**				**X**	**X**	**X**		F,L,S	Anti- inflammatory, analgesic, astringent, febrifuge and diuretic.	[14]
Verbenaceae	*Aloysia triphylla* Royse	Cedrón	FT1240		**X**				**X**		**X**	**X**	F,L,S	Anti- inflammatory anti-spasmodic, anti-neuralgic, analgesic, cardiotonic, digestive and chest and stomach tonic.	[14, 32]

*F* flower, *Fr* fruit, *I* Inflorescence, *L* leaf, *P* petal, *R* Root, *S* stalks, *Se* seed, *W* whole plant

The HRCHs were prepared according to the traditional methods of Loja, the plants were boiled for 10 min in distilled water and then cooled to an ambient temperature. They were then placed in a laminar flow cabinet and transferred to sterilized glass vials. The samples were freeze-dried in a lyophilizer LABCONCO model 7,754,047 for approximately 48 h. Finally, the samples were stored at 4 °C.

### Phytochemical screening

Phytochemical screening showed the presence of secondary metabolites (alkaloids, terpenoids, flavonoids, tannins, saponins, steroids, and quinones), primary metabolites (proteins, carbohydrates, fats), and oils using standard procedures [41].

### Analysis of total phenolic content

The total phenolic compounds were measured spectrophotometrically on a microplate reader (EPOCH 2 BioTek, BioTek Instruments Inc., Highland Park, Winooski, USA) according to the Folin-Ciocalteu’s method. This was applied to a 96-well microplate assay using gallic acid as a calibration standard [42]. A 50-μL aliquot of the different concentrations of HRCHs samples and standards were added to 150 μL of freshly prepared Folin–Ciocalteu reagent (1:4 *v*/v in distilled water) in a 96-well TR5003 cell culture plate (TrueLine, USA). After 10 min at 37 °C, 50 μL of the saturated solution of sodium carbonate was added to each well, and the plate was incubated for 10 min at 37 °C. The absorbance of each solution was measured at 725 nm on a microplate reader (EPOCH 2 BioTek, BioTek Instruments Inc.). The standard curve was linear between 2 and 0.03 mM gallic acid solution. The total amount of phenolics was calculated as mg gallic acid equivalent (GAE)/g of sample.

### DPPH radical scavenging assay

The DPPH free radical scavenging activity was evaluated on a microplate analytical assay according to the literature [42, 43]. A 30 mL aliquot of the different concentrations of freeze-dried samples and standard were added to 270 μL of DPPH in methanol solution (100 μM) in a 96-well microplate assay. After incubation at 20 °C for 60 min, the absorbance of each solution was determined at 515 nm using a microplate reader (EPOCH 2 BioTek, BioTek Instruments Inc.).

### Ferric reducing antioxidant power (FRAP) assay

A FRAP assay was performed according to the literature [42, 44]. This was applied to a 96-well microplate assay while monitoring the reduction of Fe^3+^-TPTZ to blue-colored Fe^2+^-TPTZ. Stock solutions of acetate buffer (300 mM) pH 3.6, FeCl_3_·6H_2_O (20 mM) and 10 mM TPTZ in 40 mM in HCl (10 mM) were prepared. The fresh working solution was prepared by mixing ten volumes of acetate buffer, one volume TPTZ solution, and one volume FeCl_3_·6H_2_O solution. This was then warmed to 37 °C before use. The FRAP working solution (270 μL) was mixed and incubated with 30 μL HRCHs/standards for 30 min in the dark. The absorbance of each solution was measured at 593 nm using a microplate reader (EPOCH 2 BioTek, BioTek Instruments Inc.). The standard curve was linear between 400 and 25 μM Trolox™ solution.

### Cells

Cerebral astrocytoma (D384) cells were kindly received from Dr. Mayra Paolillo at the University of Pavia. Colon cancer (RKO) were kindly received from Dr. Patricia Ostrosky at the Universidad Nacional Autonoma de Mexico. Prostate cancer (PC-3), breast cancer (MCF-7), lung cancer (A-549) and Chinese Hamster Ovary (CHO-K1) cells were purchased from the American Tissue Culture Collection (ATCC, Manassas, VA, USA).

Immortalized Chinese Hamster Ovary (CHO-K1) cells were cultured in HAM-F12 medium. The other cells were cultured in RPMI medium. These media were supplemented with 1% antibiotic-antimycotic, 2 mM L-glutamine, and 10% FBS. The cultures were maintained at 37 °C in a humid atmosphere at 5% CO_2_. The doubling times of the PC-3, MCF-7, RKO, and A-549 cells were established as 24 h; the CHO-K1 and D-384 cells were 16 h.

Human peripheral blood lymphocytes (PBL) were isolated by density gradient centrifugation with lymphocyte separation medium from whole peripheral blood from three donors. All donors gave informed written consent to use human blood samples for research purposes (View Ethics approval and consent to participate). The donors had the following characteristics: below age 30, healthy, non-smokers, and with unknown exposure to genotoxic chemicals or radiation. PBL were cultured at 37 °C and 5% CO_2_ in RPMI-1640 supplemented with 0.5% phytohemagglutinin; 1% antibiotic antimycotic, 2 mM L-glutamine, and 10% FBS. The cultures were maintained at 37 °C in a humid atmosphere at 5% CO_2_.

### Cell viability assay

Cell viability was analyzed with a MTS assay to assess the viability and/or the metabolic state of the cancer cells based on mitochondrial respiratory activity. A total of 5 × 10^3^ cells were seeded into each well of 96-well plates and allowed to adhere for 24 h. After 24 h, the cells were treated with freeze-dried HRCHs at 50 μg·mL^−1^. Each concentration/assay was performed in triplicate. Negative control cells were treated with the DMSO vehicle to a final concentration of 0.1% *v*/v; 0.5 μM doxorubicin was used as a positive control. The cells were then incubated with the treatments for 48 h. After 48 h, MTS (5 mg·mL^−1^) was added, and the cells were further incubated for 2 h in the dark at 37 °C. Absorbance was measured at 492 nm versus a reference wavelength of 650 nm. In cell lines with a inhibition percentage over 30%, doses of 15, 45, 60, 75 and 100 μg·mL^−1^ of each HRCHs were applied, and the MTS assay was used to measure the inhibitory concentration 50 (IC_50_) using non-linear regression.

### Annexin V-FITC/PI apoptosis assay

We next quantified apoptotic cells using an Annexin V-FITC/PI apoptosis detection kit [45]. In brief, the D384 cells were seeded in 6-well plates at a density of 1 × 10^5^ cells/well. After treatment at the IC_50_ for 48 h, both adherent and floating cells were harvested and stained with Annexin V-FITC/PI according to the manufacturer’s procedure. The samples were analyzed with fluorescence microscopy and an excitation wavelength of 475 nm (Zeiss Axioskop 2 plus); 200 cells were counted.

### Western blotting analysis

Total protein extraction, quantification, and immunoblots were performed as described [46]. Briefly, 50 μg of total protein were separated by 12–15% SDS-PAGE and transferred to a PVDF membrane (IPVH00010, Immobilon-P, 0.45 μm, Millipore). Rabbit polyclonal antibodies against Bcl-2, Bax, pS46-TP53, p-73, and pY99-TP73 were used with mouse poyclonal antibodies against pS139-H2AX, p21, and β-tubulin and a goat polyclonal antibody against H2AX; all antibodies were used at the dilution factor recommended by the manufacturer with the appropriate goat anti-rabbit, goat anti-mouse, and donkey anti-goat horseradish peroxidase-conjugated immunoglobulins (1:5000). Immunoreactive bands were visualized using a chemiluminescence kit Luminata Crescendo Western HRP substrate. Quantitative analysis of the proteins used a C-Digit Blot Scanner (LICOR).

### Measurement of reactive oxygen species (ROS)

The detection of ROS used 2′7´-dichlorodihydrofluorescein diacetate (DCHF-DA) [47]. Previously, CHO-K1 cells were cultured in 96-well multi-plates in HAM-F12 culture medium supplemented for 16 h. The cells were incubated with H_2_DCFDA (20 μM) and HBSS for 30 min at 37 °C. The cells were exposed to doses of 1000 μg·mL − 1 of the HRCHs and/either H_2_O_2_ (5 mM) as a positive control; HBSS was applied as a negative control. The multiplate was incubated for 2 h at 37 °C. DCFDA fluoride was measured after exposure on a Fluoroskan Ascent (Thermo Fisher Scientific) fluorometer at a λ_ex_ of 485 nm and λ_em_ of 528 nm.

### Alkaline comet assay

The alkaline comet assay was performed as reported elsewhere with minor modifications [48]. After 16 h of culturing the CHO-K1, two experimental conditions were performed. The first was only HRCHs (1000 μg·mL^−1^), and the second was concurrent treatment for 16 h of HRCHs (1000 μg·mL^−1^) and H_2_O_2_ (5 mM). The CHO-K1 cell cultures were mixed with 150 μL low-melting agarose (0.5%) and added to microscope slides. The slides were immersed in a cooled lysing solution (10% DMSO, 1% Triton X-100, 2.5 M NaCl, 100 mM EDTA, and 10 mM Tris) for 12 h at 4 °C (pH 10). All steps after lysis were performed in the dark to prevent additional DNA damage by another factors. All slides were submerged in electrophoresis buffer solution (300 mM NaOH and 1 mM Na_2_EDTA) (pH 13) for 20 min; this allowed the DNA to unwind. Electrophoresis conditions were 25 V and 300 mA for 20 min. A buffer solution was used to neutralize the slides after electrophoresis, (0.4 M Tris, pH 7.5). Each slide received EtBr (60 μL, 30 μg·mL^−1^) with a cover glass. The length tail of DNA damage was estimated using a ZEISS-Axioskop 2 plus fluorescence microscope, and 100 cells were scored on each slide and confirmed with Comet assay VI software.

### Micronucleus assay by cytokinesis-block (CBMN assay)

The CBMN test was performed as reported elsewhere with minor modifications [49]. After 16 h of culturing CHO-K1, three experimental conditions were performed. The first was only HRCHs and cytochalasin B at a final concentration of 2 μg/ml. The second was 24 h with HRCH, mytomicin C (MMC), and cytochalasin B. The last was MMC for 3 h followed by HRCHs and cytochalasin B. After 16 h of incubation, the cells were fixed in cold (−20 °C) ethanol 96° for 5 min washed with distilled water, re-fixed in acetic acid:methanol (1:3 *v*/v) for 5 min, and washed. The fixed cells were stained with a drop of DAPI (4′,6′-diamidino-2-phenylindole HCl, Serva), dissolved in Tris-buffer (pH 7.5) at 2 μg/ml. The preparations were evaluated with an Axioskop 2 plus microscope (Zeiss, Germany) equipped with a fluorescence illuminator (HBO50 lamp); the filter set was 365 nm excitation and 420 nm emission (specific for DAPI). Phase contrast was also used, and analysis was performed at high magnification for ~400 Å resolution. To facilitate discrimination of micronuclei (MN) and differentiation between mononucleated, binucleated (which divided once after exposition), and polynucleated cells (which divided twice or more). Scoring was performed blindly. The number of binucleated cells per dish was evaluated after scoring 2000 consecutive cells distributed as a monolayer according to the criteria described previously [50].

### Statistical analysis

All data were reported as the mean ± SEM of independent experiments. The statistical significance was obtained with one-way analysis of variance (ANOVA) followed by a Dunnet post-test; treated cultures were compared to controls. Statistical analyses were carried out in GraphPad Prism 4 (GraphPad Software, San Diego, CA).

## Results

The results of phytochemical screening tests on freeze-dried HRCHs samples revealed the presence or absence of main secondary metabolites and other phytochemicals based on the presence or absence of expected color changes (Table 2). The freeze-dried samples contained saponins, flavonoids (except HRCH 1), terpenoids (except HRCH 1), quinones (except HRCH 7 and HRCH 8), and alkaloids (except HRCH 2, HRCH 3 and HRCH 5).

**Table 2:** Preliminary phytochemical studies of HRCHs

Test	Sample								
	HRCH 1	HRCH 2	HRCH 3	HRCH 4	HRCH 5	HRCH 6	HRCH 7	HRCH 8	HRCH 9
Proteins	–	–	–	–	–	–	–	–	–
Carbohydrates	–	–	–	–	–	–	–	–	–
Fats	–	–	–	–	–	–	–	–	–
Alkaloids	+	–	–	++	–	+	++	++	++
Terpenoids-Steroids	–	+	+	+	+	+	+	+	+
Flavonoids	–	+	+	+	+	+	+	+	+
Saponnins	+	+	+	+	+	+	+	+	+
Quinones	+	+	+	+	+	+	–	–	+
Tannins^a^	–	–	–	–	+	–	–	+	–

+++ = Very positive, ++ = Strong positive, + = Fair positive, − = Absent

^a^Type: pyrocatechol

The antioxidant capacity was measured because of the presence of flavonoids and quinones in almost all HRCH samples. The total phenolic contents (TPC) of the HRCHs were initially estimated as mg GAE/g of sample by employing the Folin-Ciocalteu method; values were between 106 and 147 GAE/g (Table 2).

The antioxidant activity of nine HRCHs were evaluated in detail via DPPH and FRAP assays (Table 3). The Trolox equivalent antioxidant capacity (TEAC) values were calculated as mmol Trolox equivalent (TE)/g of freeze-dried HRCH to confirm the high antioxidant capacity of seven of the nine HRCHs samples (~1000 mmoles TE/g of freeze-dried) except HRCH 4 and HRCH 6. The ferric ion reducing capacity of the nine HRCHs was evaluated with total antioxidant capacity values ranging between 71 and 110 mmol TE/g of freeze-dried samples.

**Table 3:** Total phenolic content and total antioxidant capacity of the HRCHs

Sample	TPC	DPPH	FRAP
	mg GAE·g^−1^	TEAC^a^ μmol TE·g^−1^	TEAC^a^ μmol TE·g^−1^
HRCH 1	106.39 ± 0.1	1382.18 ± 27.7	71.35 ± 3.1
HRCH 2	147.32 ± 1.1	1126.91 ± 43.3	105.51 ± 1.9
HRCH 3	143.40 ± 2.3	999.64 ± 32.0	101.65 ± 0.4
HRCH 4	128.39 ± 2.2	230.96 ± 11.8	85.59 ± 2.0
HRCH 5	136.80 ± 2.2	858.33 ± 37.9	96.82 ± 0.6
HRCH 6	143.03 ± 1.1	584.23 ± 1.3	103.23 ± 0.9
HRCH 7	138.17 ± 2.3	1092.36 ± 69.1	99.66 ± 6.9
HRCH 8	147.31 ± 2.2	1109.79 ± 31.9	110.34 ± 1.4
HRCH 9	130.33 ± 1.1	919.64 ± 46.6	86.21 ± 1.3

^a^TEAC = Trolox equivalent (TE) antioxidant concentration

We used a MTS assay to evaluate the inhibitory activity of HRCHs on cell growth to determine whether HRCHs could affect tumor cell survival. There was no inhibitory effect on cell growth with PBL and CHO-K1 at 50 μg·mL^−1^ (Table 4). None of the HRCHs inhibited growth by more than 30% in A-549, RKO, MCF-7, and PC-3 cell lines. In D-384 cells, five (HRCH 1, HRCH 2, HRCH 7, HRCH 8, and HRCH 9) of the 9 HRCHs generated cell inhibition greater than 30%.

**Table 4:** Cytotoxic effect of the HRCH*s* on cells exposed to 50 μg·mL^−1^

	Percentages of inhibition ± (SEM)						
	Tumor human					Immortalized cell	Normal Human cell
	Lung	Astrocytoma	Breast	Prostate	Colon		
Sample	A-549	D-384	MCF-7	PC-3	RKO	CHO-K1	PBL
HRCH 1	1.7 ± 1.2	36.7 ± 4.0 ^(***)^	5.1 ± 1.0	14.7 ± 5.4	2.8 ± 0.7	N.A.	N.A.
HRCH 2	1.9 ± 2.1	40.8 ± 14.3 ^(***)^	7.1 ± 1.3^(*)^	26.9 ± 6.6 ^(**)^	3.9 ± 2.12	N.A.	N.A.
HRCH 3	1.6 ± 0.5	3.3 ± 1.6	0.3 ± 1.7	N.A.	0.4 ± 1.3	N.A.	N.A.
HRCH 4	N.A.	2.0 ± 1.7	2.7 ± 1.8	N.A.	N.A.	N.A.	N.A.
HRCH 5	N.A.	2.5 ± 1.7	4.6 ± 1.7	N.A.	N.A.	N.A.	N.A.
HRCH 6	2.4 ± 4.6	N.A.	0.2 ± 1.2	N.A.	N.A.	3.0 ± 0.3	N.A.
HRCH 7	N.A.	30.0 ± 0.7 ^(***)^	N.A	N.A.	4.1 ± 2.2^(*)^	N.A.	N.A.
HRCH 8	5.1 ± 12	32.6 ± 5.0 ^(***)^	6.9 ± 0.08^(*)^	N.A.	0.5 ± 1.5	N.A.	N.A.
HRCH 9	4.4 ± 4.7	60.7 ± 0.04 ^(***)^	0.7 ± 0.2	2.2 ± 9.6	N.A.	N.A.	N.A.
Doxorubicin (0.5 μM)	86.9 ± 6.2^(***)^	76.1 ± 2.6^(***)^	59.1 ± 7.4^(***)^	71.5 ± 1.5^(***)^	53.4 ± 3.6(***)	8.5 ± 0.3	N.A

*N.A.* No activity. Each data is given as the mean and its standard error (SEM) of at least three independent experiments. Data were analyzed by repeated ANOVA followed by Bonferroni post-test. Symbols denote statistically significant differences: *p < 0.01, **p < 0.001, and ***p < 0.0001 with respect to control and between HRCH

The data in Table 5 show the IC_50_ of the active HRCHs in D-348 cells. The IC_50_ range of effective HRCHs was 41 to 122 μg·mL^−1^. The least potent was HRCH 8 with 122.7 ± 4.3 μg·mL^−1^, and the most potent was HRCH 9 with 41.5 ± 6.2 μg·mL^−1^.

**Table 5:** Half maximal inhibitory concentration (IC_50_) of HRCHs on D384 cells

Sample	IC_50_ ± SEM μg/ml
	Astrocytoma (D-384)
HRCH 1	74.2 ± 0.7
HRCH 2	98.7 ± 7.4
HRCH 7	71.6 ± 1.8
HRCH 8	122.7 ± 4.3
HRCH 9	41.5 ± 6.2
Doxorubicin	0.1 ± 0.2

Each data is given as the mean and its standard error (SEM) of at least three independent experiments

**Fig. 1:**
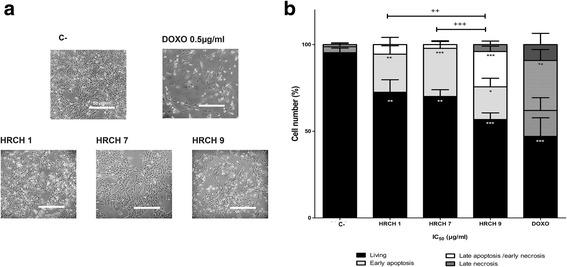
HRCH modulated morphological changes and induced apoptosis on D384 cells. **a** D384 cells were treated with control (C-), doxurrubicin (0.5 μM), HRCH 1 (74 μg·mL^−1^), HRCH 7 (71 μg·mL^−1^), and HRCH 9 (41 μg·mL^−1^) for 48 h. These were visualized under a microscope and imaged. Magnification: 40X. **b** Fraction of viable, apoptotic, and necrotic D384 cells treated with IC_50_ of HRCH for 48 h. Data are presented as mean ± SEM, *n* = 6. Data were analyzed by repeated ANOVA followed by Bonferroni post-test. Symbols denote statistically significant differences: * *p* < 0.01, ** *p* < 0.001 and *** *p* < 0.0001 with respect to C-; ^+^ *p* < 0.01, ^++^ *p* < 0.001 and ^+++^ *p* < 0.0001 between HRCHs

**Fig. 2:**
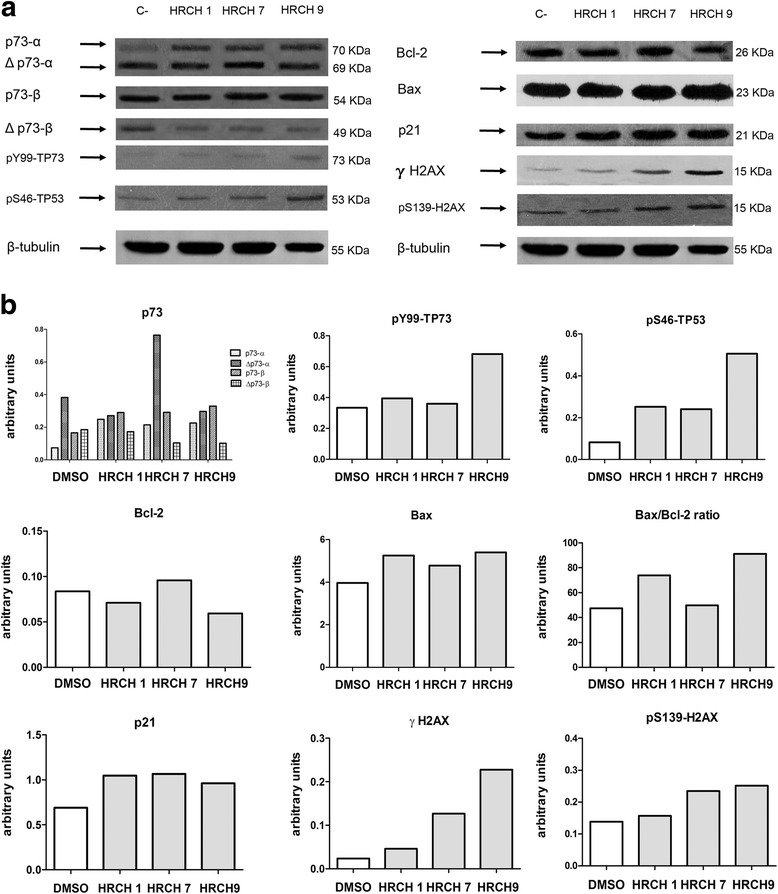
HRCHs modulated the expression of proteins on D-384 cells. D-384 cells were exposed to IC_50_ of HRCH 1, HRCH 7, and HRCH 9 for 48 h. **a** Total protein lysates were separated by 12–15% SDS-PAGE followed by western blot analysis with the indicated antibodies against p-73, pY99-TP73, pS46-TP53, Bcl-2, Bax, p21, γH2A.X, pS139-H2A.X and β-tubulin. **b** Quantitative analysis with software C-Digit Blot scanner. β-Tubulin was used as a loading control for total cell extracts

We continued studies of the cytotoxic effect produced by the most prominent HRCHs whose IC_50_ are less than 80 μg·mL^−1^ (HRCH 1, HRCH 7, and HRCH 9). The D384 cells treated with negative control and IC_50_ of HRCHs for 48 h are shown in Fig. 1a including the morphological changes. Figure 2b shows fluorescence microscopy data for D384 cells using the Annexin V-PI assay. We observed a decrease in the percentage of viable cells as well as an increase in the percentage of cells in early apoptosis: HRCH 1 resulted in ~22% inhibition, HRCH 7 was ~27% and HRCH 9 was ~19%. In cells exposed to HRCH 9, there was an increase in the percentage of cells in late apoptosis (~21%) compared with control cells (~1%) (Fig. 1b). HRCHs can modulate proteins related to death by apoptosis after evaluating Bax (pro-apoptotic) and Bcl-2 (anti-apoptotic) proteins. In both HRCH 1 and HRCH 9, there was an increased Bax/Bcl-2 ratio with respect to the negative control. Both Bax and Bcl-2 increased in cells exposed to HRCH 7 (Fig. 2a-b).

Accumulating evidence shows that p53 family members (p53, p63, and p73) play a fundamental role in the regulation of cell cycle arrest, apoptosis, autophagy, and metabolism in tumor cells exposed to stress-induced and DNA damaging agents of various origins [51–54]. Therefore, we examined whether HRCHs induced cell death and were implicated in the expression of these transcriptional factors in D384 cells. Fig. 2a-b show increased expression of p-73α and p-73β in all HRCHs (HRCH 1, HRCH 7 and HRCH 9) as well as a decrease in ΔNp-73 except for HRCH 7. It is also clear that this effect is modulated by phosphorylation of p53 when HRCH 1 and HRCH 7 are applied. In all cases, p21—a cell cycle arrest indicator—is activated. Fig. 2a-b increase with respect to the control of histones (γ-H2AX and p-γ-H2AX) related to DNA.

**Fig. 3:**
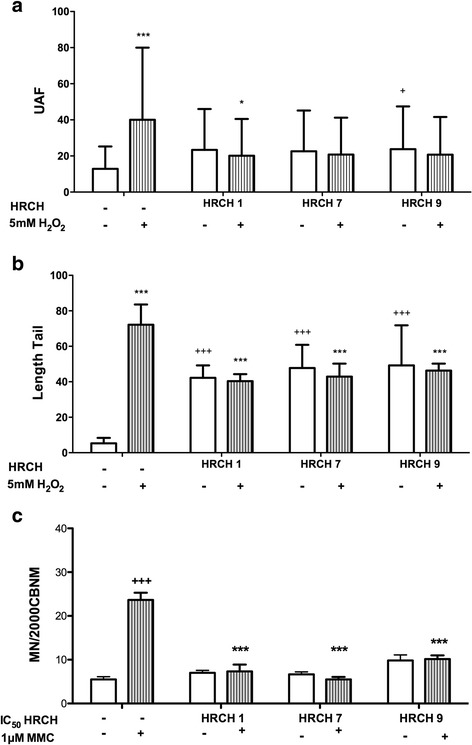
Production and inhibition of ROS, genotoxic, and anti-genotoxic activity on CHO-K1 cells exposed to HRCHs. **a** Reactive oxygen species of HRCHs with HRCHs (1000 μg·mL^−1^) and inhibition of ROS production with HRCHs (5 mM H_2_O_2_ + 1000 μg·mL^−1^). AUF: Arbitrary units of fluorescence. **b** Comet assay measured with tail length exposed to 1000 μg·mL^−1^ HRCHs and anti-genotoxic activity exposed to co-treatment with HRCH (1000 μg/ml) + 5 mM H_2_O_2_. **c** Number of Mn for 2000 BNC to 1000 μg·mL^−1^ HRCHs as well as anti-genotoxic activity exposed to co-treatment with HRCH μg·mL^−1^ + 1 mM MMC. The data are presented as the mean ± SEM, *n* = 6. Data were analyzed by repeated ANOVA followed by Bonferroni post-test. Symbols denote statistically significant differences: +++ *p* < 0.0001 with respect to control (C-) and between HRCH samples. Symbols denote statistically significant differences: * *p* < 0.01, ** *p* < 0.001, and *** *p* < 0.0001 with respect to H_2_O_2_ and between HRCHs

After observing DNA damage in tumor cells, we next studied if it occurred in normal cells using CHO-K1 cells serve as a model. We first studied the induction of ROS and subsequent genotoxicity damage and genotoxic and antigenotoxic activities. The CHO-K1 cells showed increased ROS (Fig. 3a), but this was ~50% lower than the values produced in a ROS-inducing agent (H_2_O_2_). Genotoxic and antigenotoxic activity were established with a comet assay and MNCB. Comet tail length was increased in CHO-K1 cells in all cases treatment with horchatas (Fig. 3b). However, relative to the H_2_O_2_ control, the DNA damage was ~ 30% (HRCH 9) and 40% (HRCH 1 and HRCH 7) lower.

**Fig. 4:**
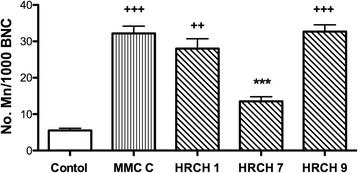
Anti-genotoxic activity on CHO-K1 cells exposed to HRCHs post-treatment with MMC. The number of Mn for 1000 BNC exposed to 1 mM MMC and 1000 μg·mL^−1^ HRCHs for 3 h. Data are presented as mean ± SEM, *n* = 6. Data were analyzed by repeated ANOVA followed by Bonferroni post-test. Symbols denote statistically significant differences: +++ *p* < 0.0001 with respect to control (C-) and *** *p* < 0.0001 with respect to MMC and between HRCHs

When we measured the genotoxic damage via the MNBN test, we observed no increase with respect to basal damage via a DNA damage-inducing agent such as MMC. The damage decreases even at basal levels (Fig. 3c) with HRCHs. To determine if it is due to a direct interaction with the genotoxic agent and the components of the HRCHs, we used the inducing agent (MMC) for 3 h followed by HRCHs. The HRCH 7 maintained the antigenotoxic effect (Fig. 4).

## Discussion

This study describes the cytotoxicity activity of five horchata mixtures (HRCH 1, HRCH 2, HRCH 7, HRCH 8, and HRCH 9) on brain astrocytoma cells (D-384). This effect is not surprising because these products are sold as a traditional “brain tonic” [2]. However, HRCHs have a high antioxidant capacity. The phenolic content is markedly higher than other plants [21] and correlates well with the high antioxidant capacity of HRCHs. Likewise, HRCHs have a strong radical scavenging ability—even better than vitamins C and E [55]. The antioxidant properties are protective against neuronal damage mediated by oxidative stress [56–59]. We emphasize that the same cytotoxic doses do not affect normal cells (CHO-K1 and PBL). This suggests that antioxidants in certain medicinal plants protect normal cells while killing neoplastic cells [60, 61]. The most potent mixtures (HRCH 1, HRCH 7, and HRCH 9) induce apoptosis, but the active ingredients are not clear because the product is a mixture. Some plants and their secondary metabolites have been previously described as inducers of apoptosis [35–59].

Post-translational modifications such as phosphorylation in the Serine 46/Tyrosine 99 of p53/P73 are critical because they facilitate binding with more affinity to pro-apoptotic proteins like Bax [54]. Their overexpression increases the Bax/Bcl-2 ratio allowing the release of cytochrome C from mitochondria to promote apoptosis [66]. Changes in protein expression are related to the percentages of early and late stages of apoptosis as can be seen in the differences between HRCH 7 and HRCH 9.

HRCHs inhibited ROS (in vitro in cells) and antigenotoxic (comet assay) and anticlastogenic (CBMN) effects. This could be due to an interaction with the genotoxic agents. However, when we tested different times between HRCHs and the alkylating agent, the anticlastogenic effect is maintained by HRCH 7 similar to various phytochemicals [62–65, 67–70]. Oxidative stress, chronic inflammation, and genomic instability events are predisposing factors for neoplastic transformation [71–73], and perturbation of these events may interfere with the malignant progression of cells. HRCH can enhance the genomic stability of cells and protect the cells against chromosomal and DNA damage induced by various genotoxic agents to gain insight into its potential use as a chemopreventive mixture.

## Conclusions

Horchata is a traditional drink in southern Ecuador that contains various medicinal plants and cytotoxic activity toward astrocytoma cells inducing regulated apoptosis in the p53/p73 pathway. Several horchatas have antioxidant and anti-genotoxic capacity that could contribute to the protective effect of this beverage. However, deeper studies are needed to discern the possible synergistic or antagonistic interactions between the species used in traditional medicine.

## Abbreviations

CBMN assay: Cytokinesis-block micronucleus assay
CHO-K1: Chinese hamster ovary
DMSO: Dimethyl sulfoxide
DNA: Desoxyribonucleic acid
DOXO: Doxorrubicin
DPPH: 2,2-diphenyl-1-picrylhydrazyl
EtBr: Ethidium Bromide
FRAP: Ferric ion reducing capacity
H_2_DCFDA: 2′-7′-dichlorofluorescin diacetate
H_2_O_2_: Hydrogen Peroxide
HBSS: Hanks’ Balanced Salt solution
HRCH: *horchata*
IC_50_: inhibitory concentration 50%
MMC: Mytomicin C
PBL: Human peripheral blood lymphocytes
PI: Propidium iodide
ROS: Reactive Oxygen Species
RPMI: Roswell Park Memorial Institute 1640 medium
TEAC: Trolox equivalent antioxidant capacity
TPC: Total Phenolic Contents
